# 

**Published:** 1889-04

**Authors:** 


					﻿Entires of the power terror at the second clean matter
Vol. IV
APRIL.1880
NO.10
DANYEL's
MEDICAL
JOURNAL
sdusbrition of the a year , in advance Singale Copies twenty five Centure
F,E DANIEL. M.D Editor, the publisher , AUSTIE,
entriee of the daniyed  tha 
				

